# Meigs syndrome in low-resource setting: a pediatric case report

**DOI:** 10.1093/omcr/omaf056

**Published:** 2025-06-27

**Authors:** Sheila Macuácua, Arlindo Muhelo, Mohammad Salgado, Agostino Calção, Helton Zucula, Ahivaldino Zita, Josina Chilundo, Sandra Mavale, Damiano Pizzol, Lee Smith

**Affiliations:** Department of Pediatric, Maputo Central Hospital, 1653 Avenida Eduardo Mondlane, Maputo, Mozambique; Department of Pediatric, Maputo Central Hospital, 1653 Avenida Eduardo Mondlane, Maputo, Mozambique; Department of Pediatric, Maputo Central Hospital, 1653 Avenida Eduardo Mondlane, Maputo, Mozambique; Department of Pediatric, Maputo Central Hospital, 1653 Avenida Eduardo Mondlane, Maputo, Mozambique; Department of Pediatric, Maputo Central Hospital, 1653 Avenida Eduardo Mondlane, Maputo, Mozambique; Department of Pediatric, Maputo Central Hospital, 1653 Avenida Eduardo Mondlane, Maputo, Mozambique; Department of Pediatric, Maputo Central Hospital, 1653 Avenida Eduardo Mondlane, Maputo, Mozambique; Faculty of Medicine, Eduardo Mondlane University, 3453 Avenida Julius Nyerere, Maputo, Mozambique; Department of Pediatric, Maputo Central Hospital, 1653 Avenida Eduardo Mondlane, Maputo, Mozambique; Faculty of Medicine, Eduardo Mondlane University, 3453 Avenida Julius Nyerere, Maputo, Mozambique; Operational Research Unit, Doctors with Africa, Rua Fernao Mendes Pinto 165, Ponta Gea 1363, Beira, Mozambique; Centre for Health, Performance and Wellbeing, Anglia Ruskin University, East Rd, CB1 1PT, Cambridge, United Kingdom

**Keywords:** Meigs syndrome, pediatric Meigs, low-resource setting

## Abstract

Meigs syndrome is a rare condition characterized by the presence of a benign fibroma of the ovary, ascites, and pleural effusion. It is very uncommon and the diagnosis is made with difficulty based on symptoms that usually mimic disseminated malignancy or tuberculosis. Although it is a benign and treatable condition, extreme presentations of late-stage diseases occur with high mortality and morbidity rates. We report on a case of a 13-year-old female presenting with misdiagnosed late-stage Meigs Syndrome in a low-resource setting.

## Introduction

Meigs syndrome (MS) is a rare and challenging condition characterized by the presence of benign ovarian neoplasm, ascites, and pleural effusion [1]. Only 1% of ovarian tumors present as an MS and it has been reported that 0.20 per 100 000 women are diagnosed with ovarian sex cord-stromal tumors [2]. Moreover, it is very uncommon before the third decade and the incidence progressively increases with age with a peak in the seventh decade [3]. The most common presenting symptoms are dyspnea, fatigue, and weight loss but considering the ovarian pathology, symptoms also mimic disseminated malignancy or tuberculosis [4]. The diagnosis is mainly clinical supported by imaging tests and, also some cancer markers such as carbohydrate antigen (CA) 125 and human chorionic gonadotropin (Beta-hCG) have been suggested, they are not specific nor sensitive [5]. By definition, MS is considered a benign condition treatable by exploratory laparotomy that is considered the gold standard treatment and also ascites and pleural effusion spontaneously dissolve after mass exercise [6]. In fact, although ascites and pleural effusion pathophysiological mechanisms have not been yet clarified, the most shared hypothesis is the venous and lymphatic one [7]. In presence of high-volume tumours, the partial occlusion of the venous return, leads to ascites. Then, it transudates through the capsule as the serous fluid [7]. The removal of mass may, therefore, resolve definitively these complications. Moreover, paracentesis and thoracentesis are a possible treatment for ascites and pleural effusion that can be performed to relief symptoms, mainly dyspnea [8]. In general, it has a good prognosis when early diagnosed but in low and middle-income countries extreme presentations of late-stage diseases occur with high mortality and morbidity rates mainly due to lack of diagnostic tools and training [6].

We report here a case of a 13-year-old female presenting with misdiagnosed late-stage MS in a low-resource setting.

## Case report

**Figure 1:**
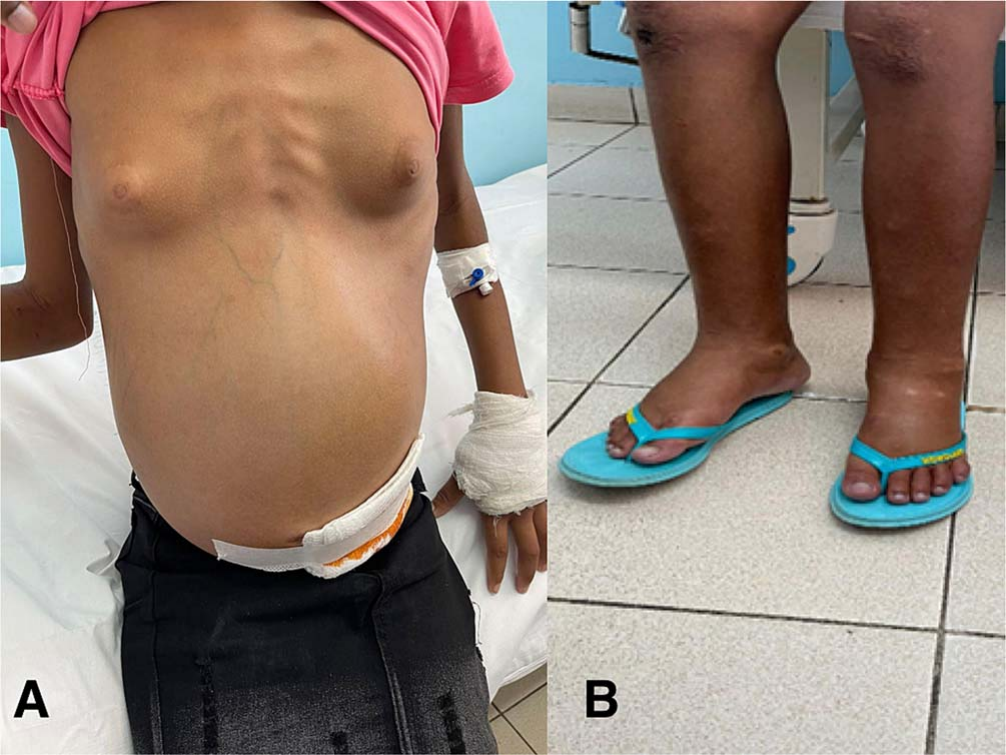
Pediatric Meigs syndrome at presentation: Tense abdomen (A) and lower limbs edema (B).

A 13-year-old female presented with an evolution of three weeks symptomatology characterized by severe and diffuse abdominal bite-like pain type, intermittent, without periodicity with abdominal distention. After one week from symptoms onset, she was treated at the local Health Center with paracetamol and ferrous sulphate without improvement. Two weeks later the condition worsened with severe chest pain in the right hemithorax associated with dry cough and difficulty breathing. Progressively, she developed weight loss, anorexia, asthenia, postprandial infarction and edema of the lower limbs and face. She was further evaluated at the local Health Center and referred to a Central Hospital with a suspicion diagnosis of heart failure, abdominal and pulmonary tuberculosis. She presented in a moderate to severe general condition, with anasarca, pale, slight jaundice in the conjunctiva, dyspnea, apyrexia and not painful cervical lymphadenopathy. She showed decreased right thoracic expansion with 2/3 decreased vesicular murmur without adventitious noises. The abdomen was tense, with positive liquid wave sign, difficult to palpate masses, painful to superficial and deep palpation (Fig. 1A). Lower limbs were affected by grade 3 edema (Fig. 1B). A thoracentesis draining of 350 ml of citrus yellow fluid was performed and resulted negative for culture, cytochemical and genexpert tests and neoplastic cell research. A paracentesis draining of 500 ml of milky ascitic liquid was performed and resulted in relief of respiratory distress and abdominal pain. An ultrasound showed bilateral pleural effusion, mainly on the right and pelvic bilateral masses. Cancer markers were negative for Alpha-1phetoprotein (1.31 U/ml) and carcinoembryonic antigen (CEA) (0.76 U/ml) and positive for CA 125 (144.5 U/ml) and Beta-hCG (2.12 U/ml). The patient was therefore admitted to the Gynecology Department. During the hospitalization she was treated with ceftriaxone and vancomycin, and a transfusion of red blood cell concentrate was performed. After 2 days her clinical conditions worsened, with significant respiratory distress, followed by cardiorespiratory arrest and death. The autopsy examination and histologic exam confirmed the presence of fibromas on both ovaries.

## Discussion

We reported a rare and extraordinary case of pediatric MS that reflects the poor social-economic context and limited resources of the context in which health care workers must work in low-resource settings. MS is, per se, a rare condition but in this case, it was even more extraordinary considering that the syndrome mainly affects women over the age of 30 while the patient was just 13 years old [9]. Proper diagnosis and management are of paramount importance for all conditions and for MS as it is considered a benign and treatable condition when treated properly and on time [10]. In very young patients as our case, CA 125 and Beta-hCG markers can play an important role in diagnostic flow, although they are not specific for this disease. Due to economic issues and transportation difficulties, the patient arrived at the referral hospital after three weeks of symptoms onset and in an already compromised general status. This initial inadequate treatment and delay in transfer demonstrates the limited resources and equipment of rural facilities and the lack of proper training and ability to recognize such an emergency. Once she presented, she was already in a critical condition, and there was limited time to stabilize her and to perform a laparotomy. There was only time to perform thoracentesis and paracentesis and relief of symptoms, but not to reverse general conditions which led to her death.

This case highlights the fragility of low-income healthcare systems mainly due to the lack of specialized and well trained health workers and equipment. For this, it is mandatory to develop effective public health policies that address these disparities and provide enhanced protection for vulnerable patients. In particular, it is crucial to promote early diagnosis both promoting patient care accessibility, both improving health workers access to diagnostic tools. In low-resource settings also telemedicine could play a crucial role in detection and management of rare conditions like S with further positive impact on all treatable diseases, especially for most vulnerable population.
